# Disease experiences of female patients with Hansen’s disease residing in settlement in Korea

**DOI:** 10.1186/s12939-020-01264-7

**Published:** 2020-08-26

**Authors:** Ho Gi Jung, Ya Ki Yang

**Affiliations:** 1 Non-Governmental Organizations, Chonnam National University, Gwangju, South Korea; 2 Department of Nursing, College of Medicine, Wonkwang University, Iksan, South Korea

**Keywords:** Leprosy, Infections, Female, Qualitative research

## Abstract

**Purpose:**

The purpose of this study was to identify female Hansen’s disease experience in settlement village in Korea.

**Method:**

For this study, 11 participants in settlement village were purposively chosen. Data were collected through in-depth individual interviews from July to December 2015. Verbatim transcripts were analyzed following Colaizzi’s phenomenological analysis to uncover the meaning of the experiences of the participants.

**Results:**

The study results showed that female Hansen’s disease experience in settlement village consisted of 9 theme and 4 theme clusters: 1) Inescapable shackles; 2) Suffered as if being in prison,; 3) In no position to be a woman or a mother; 4) Another hometown.

**Conclusion:**

The findings of this study recommends that health care professionals should pay attention not only to leprosy patients to reduce their physical and psychological suffering but also to the community and public culture to promote integration of Hansenin in the community, continued promotion and reform are needed to overcome the stigma. The results of the present study can help us in a better understanding of various aspects of female patients with Hansen’s disease residing in settlement.

## Introduction

Hansen’s disease is a disease in which the patient is infected by *Mycobacterium leprae* that infiltrates through the skin or respiratory tract. Currently, in Korea, approximately 99% of the general population has immunity against *M. leprae*, making the likelihood of infection extremely low, and even those who become infected could be treated with multidrug therapy (MDT) [1]. However, patients with Hansen’s disease (*hereinafter* Hansenin) face extreme prejudice and misunderstanding to the point of being called lepers due to the physical disfigurement that occurs as the disease progresses. Moreover, leprosy does not only influence the lives of former patients, but also the lives of their direct contacts, such as family members, friends and people in their community [2]. Stigma surrounding Hansen’s disease has a significant and persistent negative impact on all aspects of life of Hansenin, including not only their interpersonal relationships, marriage, employment, and education, but also social participation, religious activities, and healthcare utilization [3].

According to an investigation on human rights of people affected by Hansen’s disease in Korea, over 70% of the members of society have a negative perception of Hansen’s disease, while 78% of the respondents stated that they do not wish to use the same public facilities, such as bath houses or barbers, with people affected by Hansen’s disease, indicating that the negative perception and bias toward people affected by Hansen’s disease persist in our society [4]. During the Japanese occupation of Korea, Hansenin were forcibly transferred to Sorok island hospital. Physical freedom of Hansenin was severely restricted by being placed in forced confinement under the guise of treatment. Even after the liberation from Japan in 1945, oppression and sacrifices under the Japanese rule remained in place and the patients were ostracized not only by government authorities, but also by society and family.

Starting from the 1960s, South Korea created a settlement village system for Hansenin those who no infectivity or test negative for *M. leprae* in Sorok island hospital, in an attempt to promote return to society from treatment facilities [5]. In the early years of these settlement villages, the livelihood of Hansenin depended mostly on farming, outside support, and panhandling, but after the 1960s, settlement villages became livestock complexes for chicken and pig farming. During the 1970s and 1980s, combining the livestock policy and settlement village relocation policy for Hansenin was considered worldwide as a model case for success. However, maintaining the settlement villages became a challenge since the 1990s due to aging of residents in settlement villages, weaken competitiveness of livestock farms, and urbanization of settlement villages near cities [4]. During this process, Hansenin faced human rights violations, such as physical clash with nearby residents and became victims of assault and arson. Some settlement villages for Hansenin closed down due to introduction of city planning regions and decreased population. According to data published by the Korean Hansen Welfare Association in 2020, 2783 Hansenin are residing in 86 settlement villages [6].

There has been a heightened interest in settlement villages for Hansenin as a result of recent interest in the human rights of minorities and community integration policies, which has led to studies on identifying the state of settlement villages within communities and establishing welfare measures [5, 7, 8]. Studies have reported that Hansenin residing in settlement villages have low self-esteem, sense of inferiority, and sense of guilt about having Hansen’s disease, despite being able to interact with the general public, and that they are hurt even more by social prejudice, while they severely restrict their own exposure out of concerns for the well-being of their family and children [7, 9]. Women can be triply disadvantaged with regard to health concerns, due to their gender, potential disabilities and the societal stigma which arises from them [8, 10, 11]. Among 301 female Hansenin, 9.3% had the experience of being forced to have an abortion, while also having experienced intrusion with respect to their choice of spouse, childbirth, pregnancy, and parental rights [4]. The policy of forcing male Hansenin to have a vasectomy, which was implemented in 1937 during the Japanese occupation, was subsequently abolished by the Korean government after the liberation from Japanese rule, but starting from 1948, it was implemented again on couples residing on the Sorok island. Women who became pregnant were forced to have an abortion as well. Such forced vasectomy and abortion policies are known to have been in effect up until 1990 in Sorok island, as well as other national sanatoriums and settlement villages. In Korean society, women with a disability face double marginalization of being a woman and being disabled [8, 9], while they are also severely limited in their social and economic activities that would allow fair treatment as their male counterparts [11]. Accordingly, there is a need for in-depth exploration and understanding of how female Hansenin have lived their lives within the confines of double marginalization that adds the minority status of being a female to the element of being a social minority as Hansenin.

Study results and records to date on Hansenin reflect a majority position on to date from an organizational perspective of hospitals. Consequently, there are limitations in understanding the lives of Hansenin who have been placed in settlement villages as a part of return to society and settlement projects. Moreover, female Hansenin are suspected to be confined to an even greater discriminating structure, but comprehensive understanding of their actual lives is still lacking, while the unique life experiences of female Hansenin residing in settlement villages have barely been revealed. Therefore, we aimed to understand how female Hansenin residing in settlement villages in South Korea have lived their lives with Hansen’s disease.

## Methods

### Study design

The present study is an inductive study that used the phenomenological research methodology by Colaizzi [12] to understand the life experiences of female Hansenin residing in settlement villages.

### Participants

We used snowball sampling and those who had problems communicating or refused to participate in the study were excluded. After visiting Hansenin-related organizations and local self-governing bodies to explain the objective of the study and to ask for their cooperation, we were introduced to candidates who may participate in the study. Considering the age, time of disease onset, period of relocation to the settlement village, and length of residency, we conducted the interviews by being introduced to other candidates who could increase the representativeness and diversity [13]. 11 female Hansenin were interviewed. The mean age of the participants was 75 years. All 11 participants had been married, with seven participants still living with their spouse and four participants living alone as a widow. Place of birth was three from Jeollanam-do, three from Jeollabuk-do, and five from Gyeongsangbuk-do. The participants had been hospitalized in Sorokdo Hospital and Aeyangwon, but were subsequently relocated to settlement villages after recovering and testing negative. Their age at the time of relocation ranged between minimum of 28 years and maximum of 45 years. The length of stay at settlement villages varied between minimum of 20 years and maximum of 60 years.

### Ethical considerations

This study was approved by the institutional review boards of Wonkwang University (IRB No. 201407-SB-041). For the ethical consideration of study participants, we provided full explanation on the study objective, procedures, and data storage and disposal methods prior to the start of the study. We also informed the candidates about anonymity of data, recordings not being used for purposes other than the present study, the right to refuse to answer specific questions, and right to withdraw participation from the study at any time. Subsequently, we received an informed written consent from those who wished to participate in the study (for those who had difficulty submitting a written consent due to disability or old age, verbal consent was obtained and recorded). The interview data collected through the study did not include personally identifiable information to assure anonymity and data were not shared with anyone outside the study to guarantee confidentiality.

### Data collection

Data were collected by face-to-face interviews, for which, we used a portable recorder with consent from the participants. Specifically, data collection was carried out by the following procedures. First, before the actual interview, we explained the study objective and how we were introduced to the participant, and then checked whether the participant can respond to the interview by telephone or personal visit. For participants who consented to the interview, we set the date, time, and place of interview and conducted the interview accordingly.

Secondly, we constructed a basic survey sheet for the interview and a list of interview questions. The basic survey sheet was designed to record information about name, date of birth, place of birth, education level, occupation, religion, marital status, family, economic status, current address, current disease history, contact information, place of interview, time of interview, interviewer, and interview situation. We used open-ended questions in the initial interview, beginning with an introductory question of “tell us about your life as a female Hansenin.” Subsequently, each participant was encouraged to voluntarily expand on her own experiences with details about the time of disease onset; personal and social perception about Hansen’s disease; disease treatment and hospitalization process; process of relocating to the settlement village; and daily life at the settlement village. Authors constructed questions based on existing literature [4, 6–8, 14, 15].

There was a total of 11 participants and we conducted one to two interviews per participant. This is because there was a difference in the subject’s health status and story content. Each interview lasted 1–2 h. The recorded interviews were transcribed, and to produce a transcript that “vividly express everything with no omission,” non-verbal situations, such as laughs, silence, and nodding were also included in the transcript. We also created an interview log about things that we experienced or felt during the interviews and entries in the log were used as analysis data. Interviews for collecting data were carried out to the point of saturation when new concepts could no longer be extracted. The interview period was between July and December 2015.

### Data analysis

Data were analyzed following Colaizzi’s [12] phenomenological analysis, in which the cyclical process of simultaneous data collection and analysis are conducted and repeated until no new information is obtained [16]. First, we recorded the statements from all participants and repeatedly read the statements to get an in-depth understanding and hidden meanings of their experiences, based on which, meaningful statements directly related to the phenomenon were extracted. Then, significant phrases and sentences that pertained to the experience of female hansenin were extracted and eliminated repetitions. And we transformed participants’ statements into a more general formulation. In the third step, we attempted to discover what was hidden in each significant statement and created formulated meanings. Then, formulated meanings were sorted into clusters of specific themes. In the fifth step, we integrated all of the results into a narrative exhaustive description. Then, we identified the fundamental structure of the experience in unequivocal statements. In the final step, we validated the study by asking the participants if the findings captured the essence of their experience. The results were described in realistic narrative style based on data that most clearly revealed the study themes [17].

### Reliability and validity

Rigor and suitability in qualitative studies indicate how much the findings in the study and the interpretation of such findings are reliable and valid. Validity of qualitative studies is inductively derived through the process of verification during theory generation, while testing the theory derived by such process is verified in the process in which continued data collection and analysis take place simultaneously [16]. To assure validity of the results derived in the present study, we continuously compared and analyzed the collected data. During this process, we repeatedly confirmed with the participants that our interpretation and depiction of their own experiences were valid. We also used text and non-text data obtained in advance for supplementing or comparing the contents of interviews. To test the content validity of the data obtained through interviews, data rearranged from responses given by the participants were divided by question items, which were read and reviewed. The contents of interviews were compared to the data that were classified and rearranged, while testing was conducted by authors with much experience in qualitative research.

## Results

9 themes, and 4 theme clusters were identified. The four theme clusters were “inescapable shackles,” “suffered as if being in prison,” “in no position to be a woman or a mother,” and “another hometown.”

### Inescapable shackles

Our society used to perceive Hansen’s disease as a form of divine punishment. When the participants in the present study first learned that they had Hansen’s disease, they experienced debilitating fear and helplessness. As the initial symptoms of Hansen’s disease set in, they experienced fear of their body becoming deformed. Although they were able to live at home with help from family, they were semi-forcibly institutionalized due to unaccepting attitude by the neighbors and desperate hope to treat Hansen’s disease, whereby they were cured. After relocating to a settlement village, they still could not go out to the outside world due to prejudice about the disease and the limitation of being a woman, living an isolated life inside the settlement village.

### Changing body and living in hiding

All participants experienced frustration and fear about the initial symptoms of Hansen’s disease, while also experiencing fear about their own body undergoing gross deformation against their will. They recognized their own disease through bodily deformation. Hansen’s disease also places a heavy burden on the family from being a Hansenin family. Hansenin hid in their own homes and their family also lived like sinners. The disease became worse from not receiving proper treatment due to being neglected and only staying inside the house. Hansenin also had the psychological burden of knowing that their disease was causing other family members to be ostracized.

*My knee became flaky one day and my elbows the next day. It did not hurt at all, but it kept leaving a scar. Back then, I had eyebrows, hands, and feet... People didn’t recognize me because my face became swollen and my appearance changed. (#2)*

*After the face became swollen, deep wrinkle-like lines formed and face became bluish and fat from being swollen... My eyes were lifted and only my eyes became frighteningly large, making me ugly. (#4)*

*When food was brought to the room, that’s all I ate and I could not go outside. If I really wanted to go out, I had to go out at night. Everyone was in a tough position because of me. My younger siblings are just as big a victim as I am. Our neighborhood used to share a common well, but they wouldn’t let us drink from the well. (#5)*

### Barriers too high to overcome

The main source of income in the settlement villages came most from agriculture and livestock farming. Couples worked together within the settlement villages, but mostly men were responsible for other social activities outside the settlement villages. Accordingly, female Hansenin lived their entire life isolated from society, half-willingly due to not wanting to expose themselves to society. Female Hansenin limited their life boundaries to the settlement villages and strictly hid themselves to be isolated from society.

*When I first relocated, it was good that I can move around freely. However, whenever I got in a car to go outside, the driver and others kept staring at me. They would take my ticket with long tongs... So, I did not like going out anymore. (#1)*

*I went to Gwangju for a medical exam. I went with my older sister and it took a whole day by boat and car. When I touched the hospital form on this side, the nurses would only grab the other side. If nurses did that, so what would others do. (#8)*

*I want to work outside, but it is too much of a hassle to go outside, so I stay here doing the laundry and feeding the pigs. (#7)*

### Suffered as if being in prison

The life at the Sorokdo hospital for treating Hansen’s disease, which was chosen semi-forcibly, was a continuation of misery due to forced labor and inhumane treatment, rather than receiving the necessary medical treatment. The primary purpose of hospitals that forcibly quarantined Hansenin was not to treat Hansen’s disease, but to maintain the health and safety of non-Hansenin. Therefore, these hospitals were not actually facilities for treating Hansenin, but were facilities for forced confinement, similar to a prison to confine them to provide a sense of safety to non-Hansenin.

### Left the family and entered a confinement facility to live

The participants left their family behind and entered confinement facilities to receive treatment for Hansen’s disease and escape from the persecution by the community. With the enactment of the Leprosy Prevention Act in 1935, this is a period during which time the isolationism policies targeting Hansenin were strengthened. This was not only forced confinement due to the isolation policies of Japan, but also hospitalization that had to be chosen to survive the social circumstances that threatened their very survival as humans. This could be viewed as forced expulsion from community and not a choice, indicating the desperate struggle for survival by Hansenin.

*I was living hidden in a small room, but the local police came and said that there is a good place where they could give me medicine and treat my disease, so I left the house with them to be hospitalized. (#9)*

*When I left the house to go to the hospital, I had many thoughts about whether I am going there to live or to die. I was leaving to treat my disease, but it was quite scary to be away from my parents. (#10)*

### Hellish life at the confinement facility

The hospitals that the participants had chosen for treatment and survival were closer to confinement facilities than treatment facilities. They were confined to Sorokdo Hospital and other inland hospital between 1944 and 1991, where they experience the tumultuous events of liberation from Japan and the Korean War. Moreover, because a majority of Hansenin roamed around the country in a life of hunger before being confined, they faced severe violence and penalties, such as brutal beatings, surveillance, and lock-ups for violation of hospital rules based on the importance of keeping order inside the hospital. In particular, it has been reported that the lock-up facilities were places where detention and punishment took place without any due legal process [18]. Despite being patients under treatment, they suffered malnutrition and injuries to their hands and feet from forced labor, which eventually left physical disabilities. All food ingredients were government-supplied, but due to constant shortage of supplies, they experienced tremendous suffering due to starvation. As mentioned, the participants had been institutionalized in the hospitals for treatment, but instead of focusing on the treatment, they experienced oppression and exploitation, while suffering malnutrition and forced labor.

*Being in captivity, there is definitely a sense of captivity tied to being on an island. Not being able to go out freely, it is suffocating. There were some people who died by drowning while trying to swim to land for freedom. There were others who got imprisoned for getting caught while trying to escape by boat at nighttime. (#3)*

*They used to say if you’re going to die, die on a Saturday since they don’t do autopsies on Sundays. When we die, they always do an autopsy, so if you just want to be cremated, then just die on Saturday. (#2)*

*There was no luxury of even thinking that I should be pretty as a woman. We had enough rich just once a month and had only five pieces of cotton wardrobes. Didn’t even have heaters and air conditioners like today. My hands became stiffer, making them more vulnerable to frostbites when it gets cold and getting burned from grabbing something hot without even realizing it. (#1)*

### In no position to be a woman or a mother

Many participants began a life of marriage against their will during their stay at the hospital, and in this process, some were deprived of their right to bear children due to vasectomy forced on their husband. Even if some gave birth against difficult odds, they lost their parental rights with their babies forcibly sent to foster care to prevent the spread of Hansen’s disease, which indicated that their entire maternal rights were violated. Because it was difficult to withstand the life of confinement as a woman, they did not have the psychological luxury to be upset or disappointed about their physical deformity. Female Hansenin faced continuation of hardship filled life, to the point of believing that being concerned about their outer appearance was just a luxury.

### Lost femininity and motherhood

The participants were married to other Hansenin at the confinement facilities through matchmaking by elders. Although male and female Hansenin were segregated inside the facilities, cohabitation was allowed starting from 1936, as long the male had a vasectomy, for the purpose of promoting mental stability of the patients and to eliminate factors that lead to discord [19]. Marriage of female Hansenin usually involved matchmaking, often being married by force against their own will. On one hand, they were married without having a say in the matter, but on the other hand, it helped them to overcome loneliness they felt during the difficult times at the hospital, while also alleviating anxiety due separation from family and providing mental stability. The relationship formed by married had a different meaning than the patient community within the hospital, and for female Hansenin, their spouse was someone to rely on, more just a typical spouse in a regular family setting.

*Some lady told me to live with that guy because I’m lonely. I got married when I was 19 years old. I didn’t know what I liked or what was good, but because I was so lonely and hungry, I lived with him with a sense of having someone to rely on. (#6)*

*We met at the hospital. I don’t know anything about dating. There were precious few women and lots of men. I just married him thinking that once I was cured, I can just go home and that will be the end. (#2)*

*Men kept asking me to marry them. But when I told them I didn’t want to, they made me kneel and forcibly... It makes me think that was not a person should live. (#10)*

### Crow in crows

Among the participants, five Hansenin who had been placed in Sorokdo Hospital were deprived of the opportunity to have children due to forced vasectomy performed on their husband. Two Hansenin who were placed in inland hospitals had experience of abortion after forceful demand from the hospital upon becoming aware of pregnancy. To justify their rationale for vasectomy based on the claim that Hansen’s disease is hereditary, the hospitals used the expression “crows come from crows” or “crows don’t give birth to magpies.” In the process, they deprived Hansenin of their rights to pregnancy, childbearing, and parenting. Among the participants, five Hansenin had experience of having children. However, once the child reached the age of 9 months, the child was forced to be separated and sent to foster care. Sorokdo Hospital provided the mothers with an opportunity to visit their child once a month, but the parents had to see their child on the road in front of the foster care facility, keeping a certain distance from the child with the wind against the child’s back and parents facing the wind. Because of so much sighing, it even took on a name of its own as “Sootanjang” [19, 20]. The participants who have children felt sorry and regretful toward their children. They were aware of the social discrimination that the children experienced while staying with their Hansenin parents.

*I have no children. My husband has a vasectomy. If I had a child inside, how did it get there? I was born a woman, but I cannot have a child, so I really not a woman. (#9)*

*Even after sterilization surgery, they would call the women and poke the stomach from time to time. They would also often ask young women when they last had their period. (#3)*

*I became pregnant and gave birth at the hospital. After 9 months, they sent my child to foster care. Afterwards, they forced me to have the surgery. They call our children “uninfected child.” They say our children haven’t been infected yet, but will eventually be infected, so we should send them away. A baby who hasn’t even been weaned off breastfeeding. (#4)*

*It was very hurtful when our children got married. We worry about the in-laws objecting to the marriage if they find out we have leprosy. We can’t even attend our children’s weddings. Uncle and aunt took seat at the parents’ place. I only saw the wedding from photographs. (#2)*

### Deformed body

Hansenin often use the expression “3 years of not knowing, 3 years after knowing, and 3 years of rotting,” which refers to the process of physical damage setting in due to not treating the disease properly immediately after the onset. All participants had some form of physical deformity due to Hansen’s disease. In particular, three participants had disabling sequelae, such as loss of fingers and toes, sunken or protruding eyes, and facial deformity. The participants believed that their bodily features could give sense of disgust and fear of transmission to non-Hansenin, and as a result, they had feelings of damnation, fear, shame, and guilt about their physical deformity. Especially as women, they felt a very significant sense of loss from falling short of the norm for femininity due their physical deformity deviating from the socially accepted standard for beauty. They attempted to reduce their deformity by tattooing their eyebrows and wearing accessory equipment, but they also suffered infection due to unsanitary procedures and associated cost burden.

*I always that I’m a woman, so I should be pretty. It bothers me that whenever I go out, people run away. (#6)*

*I had my eyebrows tattooed so I can go out into society, but the wound got infected and the scar is even worse than before. (#1)*

*I can’t even see now, so I fall two, three times just trying to stand up. I can’t even grab things with my hands. It’s difficult without someone helping me. (#8)*

### Another hometown

The participants became afflicted with Hansen’s disease due to an unavoidable fate and lived a subhuman life that is hard to put into words. However, they are now accepting their spiteful life with feeling of letting everything go. Moreover, they were waiting for death that would put an end to their suffering. Participants who had attempted suicide before were resigned to live out their life with a sense of hopelessness that they could not even die as they had intended. Meanwhile, some reflected on their past life filled with suffering just to survive and were living the rest of their life with prayers with the help of religion.

### Backroad of life stained with suffering

Starting from the late 1940s, the movement to settle vagrant Hansenin began, and by the early 1950s, many settlement villages had been established. During this period, many of the study participants were relocated to settlement villages. However, the isolation policy of the settlement village system still blocked social integration and recreated invisible discrimination and ostracization. Physical deformities caused by Hansen’s disease were affirmed by the views of others and Hansenin felt sense of self-guilt and unfairness about having to suffer a divine punishment as a sinner without having sinned. Moreover, they could not fight back against social prejudice, but lived with feelings of hatred, anger, resentment, grief, and sadness. Even after relocating to settlement villages, female Hansenin lived as they intentionally isolated themselves from society.

*People didn’t do anything at first, but they would move away after seeing my face. Even if some people got angry at me, I couldn’t say a word even though it was so unfair. I’ve lived to this point with my head down. (#7)*

*I went out into the main society, but I couldn’t live there. People don’t say they like it or don’t like it, but I think to myself about me being a leprosy patient. (#8)*

### Self-healing at second sanctuary

The participants not only faced institutional discrimination and ostracization for a long time, but they were also reluctant in revealing their own lives and history. The fate of physical presence with erased voice, the thing that would help overcome this above all would be restoration of their sense of pride that their lives are valuable. They shared the common traits of leaving their original family and being placed in confinement facilities for treatment, whereby they overcame Hansen’s disease and lived in settlement villages, cutoff from the outside world. They perceived each other as precious beings with greater meaning than just family and spouse. Instead of exposing their negative experiences and memories of painful past, they appeared to adopt strategies for minimizing negative experiences in enclosed and isolated space and maintaining psychological stability and freedom. Despite social isolation that had persisted for a long time, the participants had accepted their disease and were living with the mindset of being thankful for the present.

*Here, we can talk about each other’s situation. In the outside world, we can’t talk about ourselves. That’s why it’s difficult to get close to others, so this place is most comfortable. (#1)*

*Here, everybody is family. We have stepsons, stepdaughters, stepparents, brothers, and sisters. We sympathize with each other. Even though my hands are like this, there is nothing to be embarrassed about. (#10)*

*My mind is peaceful and happy. I don’t resent anything now. If things stay the way they are and my time comes, I would be satisfied. I have nothing more to wish for. (#3).*

## Discussion

The objective of the present study was to understand how female Hansenin residing in settlement villages in South Korea have lived their lives with Hansen’s disease. The results showed that female Hansenin were living a life of seclusion after contracting a dreadful disease called Hansen’s disease, after which, they were placed in confinement facilities for treatment purpose and subsequently relocated to settlement village upon completion of treatment, where they built a new family. However, they were still living while accepting the spiteful life of persistent social discrimination and limitations as a woman. They were thoroughly deprived of their life as a woman and lived a life full of experiencing social isolation and discrimination as a patient with Hansen’s disease. Moreover, unlike male Hansenin who could not avoid social activities due to economic activities, they took on a survival method of limiting their life strictly to the settlement village and living in seclusion, showing a more timid and passive lifestyle. The implications derived based on the findings in the present study were as follows.

Participants regarded their disease as a divine punishment. The participants all experienced embarrassment, shame, and sense of stigma from changes occurring to their body in the early stage of Hansen’s disease. They stayed in seclusion at home to avoid social persecution. This was similar to the lives of patients with Hansen’s disease in Taiwan and Nepal who attempted self-treatment at home or in seclusion before being diagnosed with Hansen’s disease [21]. Reported causes and determinants of stigma related to leprosy are the external manifestations of the disease, cultural and religious beliefs, fear of transmission, association with people considered inferior and public health-related interventions [3]. Females are even more vulnerable among Hansenin, experiencing even higher level of isolation, loss of relationships, and ostracization than males with similar severity of Hansen’s disease. Therefore, they were victimized even more from Hansen’s disease and stigma associated with the disease [22].

Today, Hansen’s disease is a treatable disease and the perception of the disease has changed, allowing patients to receive treatments from home. However, Hansenin only have scarring from the disease and disability, but the sequelae from prejudice, discrimination, and bad isolation policy, such as forced sterilization surgery, have not disappeared from society, even today. This reflects the fact that even if discrimination against Hansenin is rectified by policy or institutional measures through legislation, the discriminatory and exclusionary attitude that has deeply set root in our daily lives still do not change easily. In fact, even health professionals who are not familiar with Hansen’s disease fear or exaggerate the risk of Hansen’s disease being contagious [7, 23]. Discrimination by medical institutions and professional has very serious social and problematic implications. Because Hansen’s disease is a disease that causes suffering for a very long time with various forms of symptoms, it desperately demands state-of-the-art treatment at general hospitals or university hospitals in major cities. Despite the operation of policies such as partner hospitals, Hansenin became indigenized to the habit of being isolated in Sorokdo or other remote locations, and thus, there is the paradox of university hospitals, which are equipped to handle Hansenin in active and professional manner, are very unfamiliar with the disease [7]. Therefore, it is necessary to guide medical institutions and Hansenin to allow Hansenin to use tertiary hospitals in major cities more actively and freely.

Even after treatment, many patients are left with deformities, sores, cracked skin, and amputated limbs [1]. In particular, female Hansenin had a greater tendency to accept the physical deformity caused by Hansen’s disease as a heavier burden than their male counterpart. To overcome the process of being stigmatized, continued promotion and reform are needed on the point that the disease is not contagious after treatment [24]. Moreover, coping skills, training of self-care skills, and consultation should be provided according to the characteristics and level of individual Hansenin [25]. Concealment of the disease due to social stigma about Hansen’s disease and Hansenin could delay diagnosis and lead to inappropriate treatment, which could cause the condition to worsen.

Female Hansenin were placed in confinement facilities for medical treatment, but they lived without being able to enjoy their femininity and motherhood. They suffered as if being in prison due to abusive treatment at confinement facilities, including surveillance and incarceration. Confinement facilities for Hansenin have the facade of places for treating Hansenin, but the fact is that they are symbols of discrimination as places of isolation and ostracization to safely protect non-Hansenin. The life in confinement facilities made it difficult for a woman to feel sorry for herself about the physical deformities that appeared in her body. To them, life was a desperate battle to survive within the strict rules within the hospital. During this process, being deprived of their motherhood was the biggest pain for these women. The findings in the present study were similar to the results of a study on Taiwanese female patients with Hansen’s disease, where those who gave birth between 1950 and 1979 were forced to give up their parental rights and give up their babies to foster care, causing them to experience guilt and grief [26].

Female Hansenin experienced difficulties in engaging in economic activities due to sequelae, such as physical disability. Most were unable to have children due to vasectomy performed on their husband, but even those who gave birth were isolated from their children, meaning they were living with no dependents. Many elderly Hansenin did not have children or were cut off from their children, and thus, there is a problem with caring for such elderly once they give up their livelihood due to disability and old age [27]. Economic difficulties could lead to problems in receiving support for medical services, while not having children could affect deficiency in emotional support. The life expectancy of Hansenin is approximately 5 years shorter than the general population. Because higher severity of physical disability is associated with higher severity of depression, accurate understanding and nursing are needed for damaged body, while there is a need for measures to improve the quality of life of and provide emotional and medical support to Hansenin without dependents [28]. In Korea, 19 Hansenin who were forced to have an abortion or sterilization surgery filed a lawsuit against the government in 2011. For the first time, the decision called for restitution by the government, recognizing the pain and attempting to accept them as members of society. Continued effort is needed at national level to find various methods to continue to soothe their pain. Moreover, support for services, such as eyebrow tattooing and artificial accessory equipment, should be considered to reduce deformities that appear in female Hansenin.

The findings in the present study showed that relocation of Hansenin from confinement facilities to settlement villages appeared as if it would give freedom to female Hansenin, but in fact, it was a form of social discrimination with imprisonment inside the settlement villages. Female Hansenin put their spiteful life behind and were living out the rest of their life by viewing the settlement villages as their second hometown. In other words, they isolated themselves in the situation of the uniqueness and relative isolation of settlement villages and showed the attitude of accepting social prejudice. Physical deformity is associated with femininity, which could cause them to experience a sense of loss. A study that found female Hansenin having negative attitude toward areas where physical damage appeared and had higher level of depression than male Hansenin supported the findings in the present study [29].

There is a need for a system that can support female Hansenin to accept their life and comfortably live out the rest of their life. Because many female Hansenin live longer than their spouse who is a Hansenin, they face death without a spouse. Moreover, regardless of whether they have children or not, they are unable to live while maintaining a close relationship with family, and thus, there is no apparatus for relieving anxiety and fear about death. Feelings such as fear, shame and low self-esteem are also experienced by those affected, and their children. Further research is necessary to ensure that mental health impact is included when determining the burden of disease for leprosy, and to relieve this burden. Female Hansenin became accustomed to the method of appeasing and accepting their spiteful life and became familiar with prolonged social discrimination and ostracization. As a result, they had the tendency of not claiming or demanding their own rights [30]. Therefore, there is the need to provide programs, such as consultation and recall therapy, to recognizing and accepting discrimination and ostracization that they have experienced. Moreover, spiritual intervention should also be considered to offer comfort during the process of preparing for death.

## Conclusion

As a result, we identified the deep fetters and meaning of female Hansenin’s life through their own voices. In addition, they currently have another overlapping minority trait of being old aged, meaning they are the subject of interest and heightened awareness by our society. We hope that the present study could be the beginning of understanding the tiresome and spiteful life of female Hansenin. It is important to emphasize that leprosy is treated in public health services, even if the person has medical insurance or private payment conditions. This may qualify the care and help in the indication of public policies for coping with diseases exacerbated by social inequality. Based on the findings in the study, we propose the following with respect to nursing research, practice, and education. First, this study examined the life experiences of only some female Hansenin residing in settlement villages. Therefore, repeated studies may be needed on female Hansenin residing in other settlement villages or at home. Second, we suggest the development of operating system and increase the number of medical personnel treating the Hansenin to increase their access to treatment in the villages. Third, as the first step in promoting integration of Hansenin in the community, continued promotion and reform are needed to overcome their stigma.

### Limitations

This study examined the life experiences of only some female Hansenin residing in settlement villages.

## Data Availability

The datasets during and / or analysed during the current study available from the corresponding author on reasonable request.
